# R229Q Polymorphism of NPHS2 Gene in Group of Iraqi Children with Steroid-Resistant Nephrotic Syndrome

**DOI:** 10.1155/2017/1407506

**Published:** 2017-04-26

**Authors:** Shatha Hussain Ali, Rasha Kasim Mohammed, Hussein Ali Saheb, Ban A. Abdulmajeed

**Affiliations:** ^1^College of Medicine, Al-Nahrain University, Baghdad, Iraq; ^2^Al-Imamein Al-Kadhimein Medical City, Baghdad, Iraq; ^3^College of Pharmacy, University of Al Qadisiyah, Diwaniyah, Iraq

## Abstract

*Background*. The polymorphism R229Q is one of the most commonly reported podocin sequence variations among steroid-resistant nephrotic syndromes (SRNS).* Aim of the Study*. We investigated the frequency and risk of this polymorphism among a group of Iraqi children with SRNS and steroid-sensitive nephrotic syndrome (SSNS).* Patients and Methods*. A prospective case control study which was conducted in Al-Imamein Al-Kadhimein Medical City, spanning the period from the 1st of April 2015 to 30th of November 2015. Study sample consisted of 54 children having NS, divided into 2 groups: patients group consisted of 27 children with SRNS, and control group involved 27 children with SSNS. Both were screened by real time polymerase chain reaction for R229Q in exon 5 of NPHS2 gene.* Results*. Molecular study showed R229Q polymorphism in 96.3% of SRNS and 100% of SSNS. There were no phenotypic or histologic characteristics of patients bearing homozygous R229Q polymorphism and the patients with heterozygous R229Q polymorphism.* Conclusion*. Polymorphism R229Q of NPHS2 gene is prevalent in Iraqi children with SRNS and SSNS. Further study needs to be done, for other exons and polymorphism of NPHS2 gene in those patients.

## 1. Introduction

Positional cloning identified NPHS2, encoding podocin, as a causative gene in autosomal recessive SRNS, including focal segmental glomerulosclerosis (FSGS) [1, 2]. NPHS2 is the predominant mutation found in children over the age of 1 year [3, 4]. NPHS2 expression is restricted to the podocyte as shown by in situ hybridization studies. About 50 NPHS2 gene mutations and variants and/or nonsilent polymorphisms have been reported and recognized as potentially being involved in proteinuria. The polymorphism R229Q is one of the most commonly reported podocin sequence variations [5–7]. A functional polymorphism of NPHS2 gene, R229Q, was associated with a late-onset nephrotic syndrome and also with an increased risk of microalbuminuria in the general population [8, 9]. FSGS associated with NPHS2 mutation is uniformly steroid resistant and generally shows poor response to cyclosporine as well [10].

## 2. Aim of the Study

The aim of this study is to determine the frequency of R229Q polymorphic site of the NPHS2 gene in children with SRNS in comparison with SSNS, to study the relation of this polymorphic site to important demographics and clinical characteristics, and to determine the risk of having SRNS in relation to R229Q polymorphism.

## 3. Patients and Methods

This was a prospective case control study which was conducted in Al-Imamein Al-Kadhimein Medical City, spanning the period from the 1st of April 2015 to 30th of November 2015. Study sample consisted of 54 children having NS. Study sample was categorized into 2 groups: patients group consisted of 27 children with SRNS. Control group involved 27 children with SSNS.

Steroid responsive NS was regarded as complete remission achieved with steroid therapy. Steroid-resistant NS was regarded as failure to achieve remission following 4-week prednisone 60 mg/m^2^ followed by three methylprednisolone pulses [1, 2].

### 3.1. Data Collected

The data collected were gender, age, age of onset of NS, steroid responsiveness, family history of NS, consanguinity, and renal biopsy if done and its report. The following laboratory investigations were performed for all children: urinalysis, plasma albumin, blood urea, and serum creatinine.

Normal values of blood urea were as follows: 1-2 y = 1.8–5.4 mmol/l; >2 y = 2.9–7.1 mmol/l. Normal values of serum creatinine were as follows: child = 27–62 mmol/l; adolescent = 44–88 mmol/l [4].

Renal insufficiency was explained by 5 stages of chronic kidney disease by estimated GFR based on serum creatinine using Schwartz formula [5].

The data about hypertension and renal insufficiency are at the last follow-up time.

Each child donated 3 ml of venous blood, collected in an EDTA-containing blood collection tube. Samples were transferred to the molecular Pathology Laboratory, Department of Pathology, College of Medicine, Al-Nahrain University.

Genetic methods included DNA extraction and TaqMan real time PCR genotyping.

### 3.2. Statistical Analysis

The SPSS version 21 was employed in this research. Chi-square test was used for comparison of frequencies and *t* tests were used for comparing means. Odd ratio was used to discriminate between alleles in patients and control.

## 4. Results

Table 1 shows the demographic characteristics of both patients and control groups. Mean age of patients was 5.253 ± 4.80 years for SRNS (range from 2 to 17) and 7.7037 ± 2.829 for SSNS (age range from 2 to 15 years), while mean age at diagnosis was 5.420 ± 4.114 years for SRNS and 4.666 ± 2.650 years for SSNS. Three age groups for age of presentation and age of diagnosis are shown in Table 1.

Older age group (12–18 years) were all SRNS at time of presentation and time of diagnosis with no SSNS children (*P* value = 0.005 and 0.038, resp.).

Correlation between gender and family history of NS of the two groups was statistically nonsignificant. The patients and control groups were compared according to their clinical data and laboratory investigations as shown in Table 2. The difference was statistically significant regarding hypertension, hematuria, renal insufficiency, mean of blood urea, and mean s. creatinine level, while no significant correlation was found with serum albumin.

Renal biopsy was performed for 21 patients; results are shown in Table 3.

## 5. Genetic Results

In Table 4, genetic results were put in form of G allele (VIC labeled) representing the wild type and A allele (FAM labeled) representing the polymorphism, and comparison between patients and control has been done.

The wild type allele was found in 16 (59.3%) and the polymorphic allele (FAM) was found in 26 (96.3%) of SRNS patients. In the control group, the wild allele was found in 11 (40.7%) and the polymorphic allele was found in 27 (100%). The difference between these readings was nonsignificant *P* value.

The genotype was divided into homozygous wild gene (G/G), homozygous polymorphic gene (A/A), and heterozygous gene (G/A). No significant statistical difference was found between the two groups regarding the 3 genotypes.

In Table 5, clinical data and disease progression of 53 children with heterozygous and homozygous polymorphic R229Q were compared. No significant correlation was found regarding mean ages at diagnosis, gender, consanguinity, family history of NS, hypertension, steroid responsiveness, renal insufficiency, and renal biopsy results.

## 6. Discussion

In the present study, males predominated in both patients (SRNS) group and control (SSNS) group with slight difference. This is similar to many other studies from different regions [11–16]. Consanguinity was found in high percentage in both groups, which is expected in this community because of social traditions. This is important to be surveyed in all nephrotic children to relate this disease to its associated genes and the type of inheritance.

Coming to the genetic results found in this study, the polymorphism R229Q was found in 26 (96.3%) of 27 children with SRNS in both heterozygous and homozygous genotypes. A lower percent of this polymorphism was found by many studies [11, 13, 15, 16]. In addition, several other studies did not report any polymorphism in SRNS [7, 8, 12, 16, 17]. The polymorphism R229Q was found 100% among SSNS group in both homozygous and heterozygous genotype, which is much less than its frequency in Landau et al. [14], who did not find any, as well as Lahdenkari et al. [18] and Caridi et al. [19].

The present results are important, because the frequency of R229Q polymorphism of NPHS2 mutation among Iraqi children is largely unknown. This is because a small number of founding individuals and a high rate of consanguineous and endogamous marriages, typical of small communities, increase genetic homogeneity and highlight susceptibility genes.

In this study, obvious genotype/phenotype correlation regarding age at diagnosis, sex, consanguinity, family history, hypertension, renal insufficiency, and renal histology in both homozygous and heterozygous patients were not observed.

Such findings were similarly mentioned by several studies [13, 17, 20, 21]. While Phelan et al. found that R229Q polymorphism was associated with early onset childhood SRNS [22], Sadowski et al. found FSGS in 68% of R229Q polymorphism [23] and Tsukaguchi et al. found R229Q polymorphism associated with late-onset SRNS [24].

This polymorphism is prevalent among Czech (12%), Spanish (3.1%), French (4.5%), Brazilian (3.1%), and Italian (3.2) [16, 25]. The highest frequency of R229Q, after Czech population, has been reported in Chileans and Argentineans (7.3%) [26]. The R229Q polymorphism has a lower frequency among Africans, African-Americans, and Asians (zero to 1.5%) [16, 27]. Tryggvason and colleagues proposed that R229Q, which is present in around 4% of European populations, is associated with an increased risk of microalbuminuria [28]. Pereira and colleagues found that p.R229Q was associated with a 2.77-fold increased risk of microalbuminuria [29].

Both SRNS and SSNS in the present study did not show a significant difference regarding the polymorphism. It seems that at least one polymorphic allele of R229Q may participate in their nephrotic process, and by this a conclusive role in steroid resistance was not reached. In order to reach a conclusive decision about the role of R229Q in the pathogenicity of nephrotic syndrome, its association with other exon mutations of the NPHS2 gene must be searched for in the same study groups. Its exact frequency in the Iraqi population needs to be determined as well, by studying the frequency of this polymorphism in normal nonnephrotic children and comparing the findings with those of affected ones. Studying other exon mutations including the most reported pathologic ones like mutations in exons 7 and 8 of the same gene is necessary to unravel the pathogenesis of SRNS in Iraqi children. Of no less importance is studying other nephrotic syndrome genes. These topics are put into consideration as future perspectives of the present work.

## Figures and Tables

**Table 1:** Distribution of the study group according to demographic data.

Data	SRNS		SSNS		*P* value
	Number	%	Number	%	
Sex					
Male	20	74.1%	17	63.0%	0.379
Female	7	25.9%	10	37.0%	0.379
Age					
1–6 years	7	25.9%	10	37.0%	0.379
6–12 years	13	48.1%	17	63.0%	0.273
12–18 years	7	25.9%	0	0.0%	0.005
Age at diagnosis					
1–6 years	17	63.0%	18	66.7%	0.776
6–12 years	6	22.2%	9	33.3%	0.362
12–18 years	4	14.8%	0	0.0%	0.038
Family history					
Positive	4	14.8%	4	14.8%	0.444
Consanguinity					
Positive	23	85.2%	16	59.3%	0.033

**Table 2:** Patient distribution according to clinical data and laboratory investigations.

Data	SRNS		SSNS		*P* value
	Number	%	Number	%	
Hypertension					
Hypertensive	16	59.3	2	7.4	0.000
Normotensive	11	40.7	25	92.6	
Hematuria					
Hematuria	9	33.3	1	3.7	0.005
No hematuria	18	66.7	26	96.3	
Renal insufficiency					
RI	8	29.6	0.00	0.00	0.002
No RI	19	70.4	27	100	
Mean s. albumin, g/L (mean ± SD)					
2.400 ± 0.889			2.525 ± 0.867		0.601
Mean blood urea, mmol/L (mean ± SD)					
6.499 ± 3.491			4.149 ± 1.405		0.002
Mean s. creatinine, mmol/L (mean ± SD)					
79.518 ± 73.150			47.696 ± 14.904		0.031

**Table 3:** Renal biopsy results of 21 patients.

	Steroid responsiveness	
	Steroid-sensitive	Steroid-resistant
Minimal change disease	3 (75.0%)	8 (47.0%)
Focal segmental glomerulosclerosis	1 (25.0%)	7 (41.1%)
Mesangioproliferative	0 (0.0%)	2 (11.7%)
Total	4	17

**Table 4:** The frequency of genotyping in patients and control groups.

Alleles	SRNS		SSNS		*P* value
	Number	%	Number	%	
G (VIC)	16	59.3	11	40.7	0.174
A (FAM)	26	96.3	27	100	0.313

Genotype					
G/G	1	3.7	0	0.0	0.313
G/A	15	55.6	11	40.7	0.267
A/A	11	40.7	16	59.3	0.174

**Table 5:** The clinical data and disease progression of 53 children with heterozygous and homozygous polymorphic R229Q.

Parameter	Heterozygous GA (*n* = 26)	Homozygous AA (*n* = 27)	*P* value
Age at diagnosis (year ± SD)	4.853 ± 3.777	5.079 ± 3.144	0.814^*∗*^
Male/female	16/10	20/7	0.328^*∗*^
Consanguinity	21	18	0.244^*∗*^
Family history	5	2	0.204^*∗*^
Hypertension	9	8	0.697^*∗*^
Renal insufficiency	5	3	0.409^*∗*^
*Response to steroids*			
*SRNS*	*15*	*11*	*0.217* ^*∗*^
*SSNS*	*11*	*16*	
Renal biopsy			
MC	6	5	0.920^*∗*^
FSGS	5	3	
MP	1	1	

*∗*: NS.
